# Ultrasound Guided Core Biopsy versus Fine Needle Aspiration for Evaluation of Axillary Lymphadenopathy in Patients with Breast Cancer

**DOI:** 10.1155/2014/703160

**Published:** 2014-02-04

**Authors:** Marie A. Ganott, Margarita L. Zuley, Gordon S. Abrams, Amy H. Lu, Amy E. Kelly, Jules H. Sumkin, Mamatha Chivukula, Gloria Carter, R. Marshall Austin, Andriy I. Bandos

**Affiliations:** ^1^Department of Radiology at Magee-Womens Hospital of the University of Pittsburgh Medical Center, 300 Halket Street, Pittsburgh, PA 15213, USA; ^2^Department of Pathology, Mills-Peninsula Hospital of Sutter Health Affiliate, 383 East Grand Avenue suite A, South San Francisco, CA 94080, USA; ^3^Department of Pathology at Magee-Womens Hospital of the University of Pittsburgh Medical Center, 300 Halket Street, Pittsburgh, PA 15213, USA; ^4^Department of Biostatistics, University of Pittsburgh, Graduate School of Public Health, A414 Crabtree Hall, 130 DeSoto Street, Pittsburgh, PA 15213, USA

## Abstract

*Rationale and Objectives*. To compare the sensitivities of ultrasound guided core biopsy and fine needle aspiration (FNA) for detection of axillary lymph node metastases in patients with a current diagnosis of ipsilateral breast cancer. *Materials and Methods*. From December 2008 to December 2010, 105 patients with breast cancer and abnormal appearing lymph nodes in the ipsilateral axilla consented to undergo FNA of an axillary node immediately followed by core biopsy of the same node, both with ultrasound guidance. Experienced pathologists evaluated the aspirate cytology without knowledge of the core histology. Cytology and core biopsy results were compared to sentinel node excision or axillary dissection pathology. Sensitivities were compared using McNemar's test. *Results*. Of 70 patients with axillary node metastases, FNA was positive in 55/70 (78.6%) and core was positive in 61/70 (87.1%) (*P* = 0.18). The FNA and core results were discordant in 14/70 (20%) patients. Ten cases were FNA negative/core positive. Four cases were FNA positive/core negative. *Conclusion*. Core biopsy detected six (8.6%) more cases of metastatic lymphadenopathy than FNA but the difference in sensitivities was not statistically significant. Core biopsy should be considered if the node is clearly imaged and readily accessible. FNA is a good alternative when a smaller needle is desired due to node location or other patient factors. This trial is registered with NCT01920139.

## 1. Introduction 

The prognosis of the newly diagnosed breast cancer patient depends on a number of factors, among the most important of which is the extent of spread of disease to the axillary lymph nodes [1, 2]. Because treatment is influenced by the presence and number of axillary lymph nodes involved, evaluation of the axillary nodes has been performed in every patient that could tolerate it after a diagnosis of invasive carcinoma [3]. In the past, a complete surgical dissection of the axilla was performed, resulting in significant morbidity, including a 30% incidence of lymphedema [4]. The development of sentinel node mapping resulted in a notable reduction in morbidity; however, if a sentinel node was positive, often not discovered until final pathologic processing done postoperatively, complete axillary dissection would be performed at a later date to assess the total number of lymph nodes involved, thus requiring a second surgical procedure and anesthesia [5–7]. A preoperative diagnosis of axillary metastasis by ultrasound guided node biopsy would streamline patient care and reduce operating room time and expense by allowing definitive breast surgery and complete axillary dissection in the same operative setting, eliminating an unnecessary sentinel node procedure. While the need for preoperative node sampling in patients with nonpalpable nodes and T1 and T2 cancers has been challenged by the ACOSOG Z 11 study [8], there are subgroups of women for whom initial ultrasound guided node sampling is desirable, including those planning mastectomy or neoadjuvant chemotherapy.

Using current high frequency transducers, the axillary lymph nodes are usually well visualized sonographically. Studies have been performed attempting to identify malignant lymph nodes by their morphology but there is overlap both in the appearance of benign and malignant lymphadenopathy and in the appearance of normal and abnormal lymph nodes, with benign causes of axillary adenopathy being relatively common [9–11]. Therefore, tissue diagnosis of axillary node status remains important when it will affect patient management. Tissue sampling of suspicious axillary lymph nodes with ultrasound guidance has been performed for many years with a fine needle to obtain aspirates for cytologic evaluation. Reports in the literature have shown that FNA is useful for evaluation of metastatic disease, with sensitivities ranging from 44 to 100%, the variability being likely due at least in part to patient selection [12–18]. Studies comparing FNA to large needle core biopsy of breast masses have shown core biopsy to be more accurate and easier to interpret than FNA [19], leading breast care specialists to wonder if that is the case for sampling of axillary lymph nodes. While it is reasonable to expect the use of a core biopsy device to obtain a larger and architecturally intact piece of tissue to increase diagnostic accuracy as compared to obtaining an aspirate of cells with a fine needle, if the only question to be answered in sampling the axillary node is whether or not metastatic carcinoma cells are present along with lymphocytes, a larger architecturally intact piece of tissue may not be required. Although core biopsy is more invasive than FNA due to the larger needle size, reports have shown that core biopsy of axillary nodes is a safe and effective procedure [20, 21]. Retrospective comparisons of FNA and core accuracy in the axilla have been performed [22, 23], but to our knowledge, only one other prospective study [24] has been published directly comparing fine needle aspiration to core biopsy of an axillary lymph node for the diagnosis of metastatic breast cancer.

The purpose of this study was to determine if there is a difference between ultrasound guided core biopsy and FNA in their ability to detect metastatic disease in the axillary lymph nodes of patients with a current diagnosis of ipsilateral breast cancer.

## 2. Materials and Methods 

### 2.1. Study Design

From December 2008 through December 2010, women with suspected or recently diagnosed breast carcinoma and at least one lymph node in the ipsilateral axilla judged to be abnormal in sonographic appearance were approached for participation in this IRB-approved, HIPIAA-compliant prospective study. 105 women gave written informed consent to undergo ultrasound guided FNA, immediately followed by core biopsy of the same node, followed by clip placement. Patients unable to consent due to language or comprehension difficulties and patients deemed emotionally too fragile to discuss the subject of metastatic disease required in the consent form were excluded. Other patients excluded themselves, not wishing to undergo a second needle procedure for research purposes. One patient was excluded due to difficulty accessing the node with a core biopsy due to its location. The outcome of percutaneous node sampling was correlated with surgical pathology from sentinel node excision or axillary dissection.

Prior to node sampling, the cortical thickness, presence or absence of a hilus, presence or absence of cortical flow, node shape, and number of nodes thought to be abnormal were recorded. A Phillips iU22 ultrasound unit (Phillips Healthcare, Andover, MA) with 12 or 17 MHz transducers was used for imaging. Criteria used in determining appropriateness for node sampling were loss of the normal hilus, abnormal shape including focal bulging of the cortex, presence of cortical flow, and cortical thickening. A strict threshold for cortical thickness was not used; a node with a cortex between 2 and 3 mm was considered suspicious if the other nodes had cortices less than 2 mm. The patients were asked to rate their pain during each procedure on a scale of 1 to 10 and were informed of which procedure was being performed. The degree of bleeding (minimal or moderate) or hematoma formation, if any, was documented.

### 2.2. Study Population

Of the 105 patients, 7 patients' percutaneous breast biopsies were unexpectedly negative for malignancy (their node biopsies were all negative also). Of the remaining 98 with breast malignancy, 3 patients with both FNA and core negative nodes were excluded due to lack of histopathologic axillary surgical correlation. Thus, 95 patients constitute the cohort for assessing percutaneous node biopsy sensitivity. We included 4 patients who did not have axillary surgery but were assumed to be true positives as they were both core and FNA positive. Time between biopsy and surgery ranged from 9 days to 10 months. However, 47 of the patients had neoadjuvant chemotherapy prior to surgery. Tumor size ranged from 6 mm to 10.7 cm, with 12 patients having tumor size greater than 5 cm. Cortical thickness of the sampled node ranged from 2 mm to over 6 mm.  Table 1 shows features of the study population.

### 2.3. Tissue Sampling Procedures

**Figure 1:**
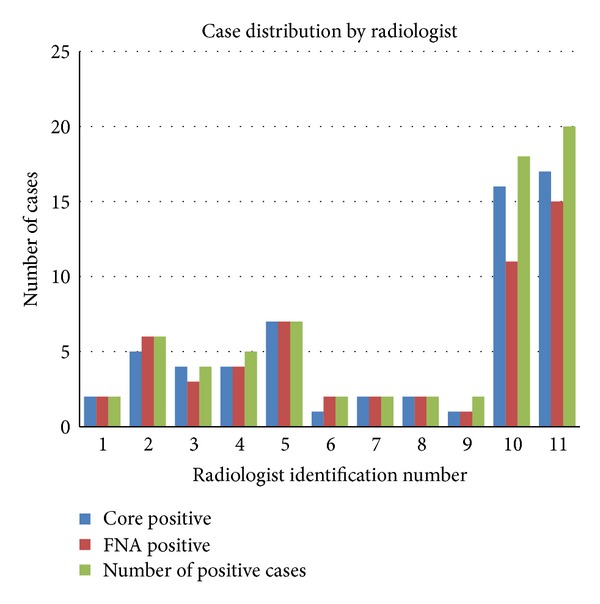
The graph illustrates the number of cases positive for malignancy for each radiologist and the number testing positive by core biopsy and FNA. (The 12th radiologist was not included, with only negative cases.)

**Figure 2:**
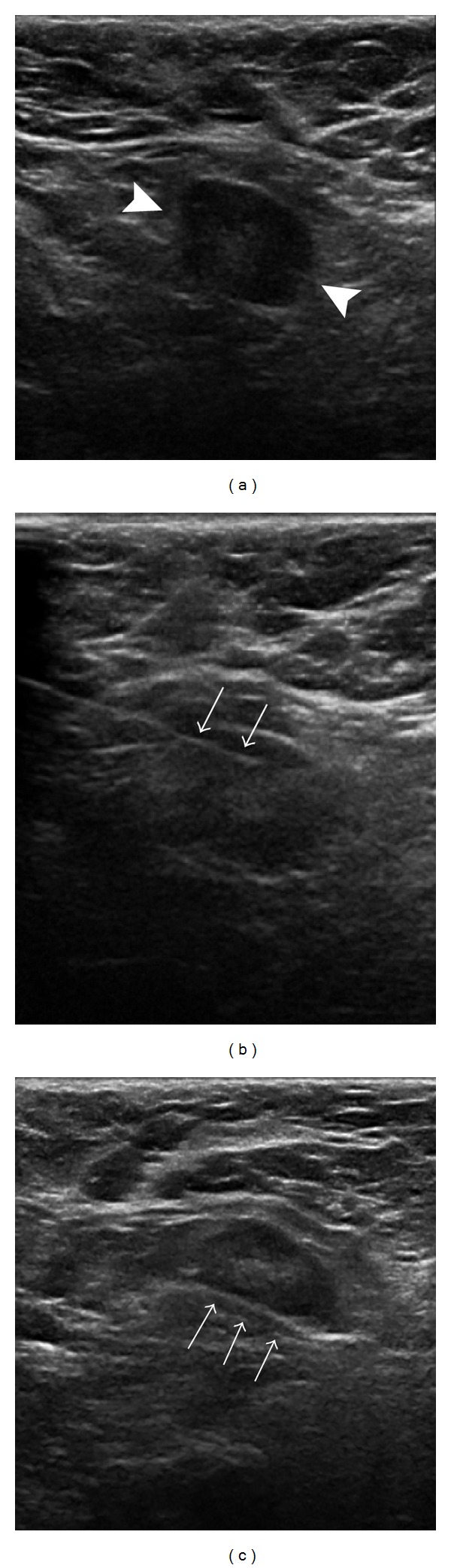
Ultrasound images of the right axilla of a 65-year-old woman with infiltrating lobular carcinoma show (a) a round lymph node (arrows) with a 5 mm cortex, (b) a 25 g FNA needle (arrows) traversing the cortex of the node, and (c) the open trough (arrows) of a 12 g core biopsy needle in the node. The FNA was single entry. The core was 1 pass. The FNA cytology was negative but the core biopsy was positive for malignancy; 7 of 18 lymph nodes were positive at axillary dissection performed less than 2 months after the biopsy.

To mimic the actual range of clinical practice, variability in sampling devices was allowed. FNA was performed using a 21 or 25 g, 2-inch needle, with one (90 cases), 2 (11 cases), or 3 (4 cases) needle entries of multiple needle excursions through the cortex of the node. The number of excursions was not recorded, but radiologists preferring more than one entry typically made fewer excursions per entry, estimated at 10 versus 20 to 30. The aspirated material was placed both on slides in 95% alcohol and in buffered Formalin for cell block preparation; a pathologist was not present at the time of the procedure to evaluate the adequacy of the samples. Core biopsy was performed immediately following the FNA with either a 14 g (86 cases), 12 g (16 cases), or 18 g (3 cases) biopsy device. The 12 g device used was Celero (Hologic, Bedford, MA). The 14 g devices were Bard Monopty, Bard Maxcore, Finesse (Bard, Tempe, AZ), and Achieve (Cardinal Health, Dublin, OH), and the 18 g devices were Achieve. One to four cores were obtained (one core in 23 cases, 2 cores in 54 cases, 3 cores in 20 cases, and 4 cores in 8 cases). With 14 g devices, only one core was obtained in 11 of 86 cases. With 12 g devices, one core only was obtained in 12 of 16 cases. With 18 g devices, 2, 3, or 4 cores were obtained. The procedures were performed by 12 different academic radiologists experienced (range 3–21 years, with over half performed by those with over 16 years of experience) in breast imaging and biopsy (Figure 1). However, for a given patient, the same radiologist performed both the FNA and the core biopsy. The cytologic material obtained was evaluated by one of three pathologists experienced in breast pathology and cytology, without the knowledge of the core biopsy result. No immunostains were used. The FNA result was categorized as negative if reported as containing suspicious cells but not actually stating that metastatic cancer cells were present. The core biopsy samples were evaluated separately by different pathologists. Suboptimal specimens were categorized as negative for both core and FNA, because the course of action in our institution in most cases would be to proceed with sentinel node biopsy rather than to repeat the percutaneous biopsy.  Figure 2 shows images from an axillary node sampling procedure.

### 2.4. Statistical Analysis

Statistical analysis was performed using SAS v.9.2. The sensitivities of the FNA and core biopsy procedures were compared using McNemar's exact test for correlated proportions. The trends in sensitivities with changing numbers of passes, entries, or needle sizes were assessed with the exact Cochran-Armitage test. Patients' subjective perception of pain levels was compared using the exact Wilcoxon sign test.

## 3. Results 

**Figure 3:**
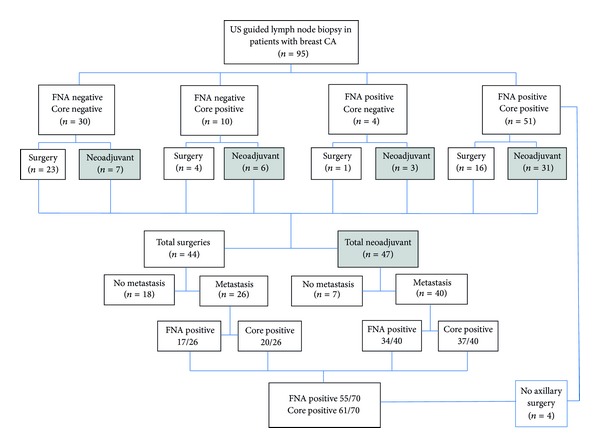
Flowchart of patients undergoing lymph node biopsy. Total surgeries = number of patients without chemotherapy before surgery. Total neoadjuvant = number of patients having chemotherapy before surgery.

Of the 95 patients in the study cohort, 70 patients (74%) had metastatic adenopathy. This group included 5 patients that were both core and FNA positive at percutaneous biopsy but were node negative after chemotherapy, 2 discordant (FNA negative, core positive or FNA positive, core negative) cases with complete pathologic response to chemotherapy resulting in negative nodes at surgery, and 4 core and FNA positive patients without axillary surgical correlation. (59 patients had positive nodes at axillary surgery.) We assumed no results were false positive.  Figure 3 is a flow chart of the procedures and results.

FNA was positive in 55/70 (78.6%) and core was positive in 61/70 (87.1%) (*P* = 0.18 (95% CI 0.032–0.166)). 65 of the 70 (92.9%) patients had a positive axillary node by tissue sampling: 51 by both FNA and core, 4 only by FNA, and 10 only by core biopsy. Thus, in this group of 70, there were 14 cases (20%) where FNA and core results were discordant (*K* = 0.3 (95% CI 0.03 to 0.58)).

The sensitivity for single pass core biopsy was 78.6% (11/14) and for multipass cores was 89.3% (50/56), which was not a statistically significant difference (*P* = 0.37). The sensitivity for the 12 g device was 89.9% (8/9), for 14 g devices was 86.7% (52/60), and for 18 g devices was 1/1; the differences in sensitivity using the 12 g “vacuum assisted” device versus other devices together were not statistically significant (*P* > 0.99). The sensitivity for 21 g single entry FNA was 76.1% (35/46) not different from 25 g single entry FNA at 78.6% (11/14) (*P* = 0.85). The sensitivity for single entry FNA was 76.7% (46/60) not statistically significantly different from multientry FNA at 90% (9/10) (*P* > 0.41) (Table 2).

The sensitivities of FNA and core biopsy were compared in Table 2 for numbers of suspicious nodes, for node hilus, cortex, and shape, and also for tumor size. FNA was the least sensitive in normal shaped nodes. FNA sensitivity was inferior to core sensitivity (*P* = 0.04) when the node hilus was visible but improved with hilar absence. Both FNA and core sensitivities improved with cortical thickness increasing beyond 2 mm, and when 3 or more abnormal appearing nodes were noted.

Of the 10 FNA negative/core positive patients, all but one had positive nodes at surgery; that patient had a complete pathologic response to chemotherapy and 20 negative nodes. One cytology specimen was reported as less than optimal, and 2 mentioned suspicious cells but were not diagnostic for malignancy. Of the 4 FNA positive/core negative cases, each performed by a different radiologist, all of the core specimens were reported as suboptimal, 2 with scant lymphoid tissue, and two with absent lymphoid tissue. In 2 of the 4 (one scant and one absent lymphoid tissue), only one core was obtained. Three of the 4 had positive nodes at surgery, and one patient had negative nodes but had a complete pathologic response to chemotherapy with neoadjuvant related changes in the axilla.

Of the 5 core and FNA negative patients with positive nodes at surgery, the clip was noted to be in a negative node in 2 cases indicating that the wrong node was chosen for sampling. The presence of a clip or evidence of biopsy was commented on in 59% (54/91) axillary surgical reports.

There was no difference in bleeding between the 2 procedures, which was minimal for all but one case that was moderate for both FNA and core. The mean pain score for FNA was 2.0 and for core was 2.4 while the range was from 1 to 8 for FNA and from 1 to 10 for core. Reported pain levels were similar during FNA and core in 63 patients (60%), greater with core in 31 patients (29.5%), and greater with FNA in 11 patients (10.5%). The higher pain level was reported significantly more frequently for core than for FNA (*P* < 0.01).

## 4. Discussion 

Our results show that although core biopsy had greater sensitivity than FNA in detecting metastasis, it did not approach statistical significance, probably primarily due to the small number of patients. These results are in agreement with the meta-analysis by Houssami et al. [22] who reported a sensitivity for FNA (24 studies) of 72.2% and sensitivity for core biopsy (4 studies) of 83.3%, which were not statistically significantly different. The only other prospective comparison study [24] reported a significantly greater sensitivity for core biopsy (88.2%) than for FNA (72.5%) but had a small sample size of 51 patients undergoing percutaneous node biopsy with axillary metastasis.

Our study included several experienced radiologists and allowed a variety of sampling devices to simulate actual clinical practice. While axillary node FNA is technically easy to perform for one experienced in image-guided procedures, the radiologist must obtain an aspirate that is both sufficient in the amount of material and at the same time not overly bloody, to enable an optimal interpretation. It is not clear why there were fewer false negative results when multiple FNA entries were performed, as the total number of needle excursions likely did not differ greatly. Perhaps the chance of obtaining a better sample was increased by using different entry sites or obtaining less blood mixed with cells from the node. The number of slides used, actual number of excursions, and length of procedure were not recorded, which could have affected the results. In some institutions, a pathologist is present when cytologic samples are obtained and can request additional sampling if the specimen is deemed suboptimal; the presence of a pathologist at the time of sampling could have improved the yield from FNA. In our institution, immunostains may be used to aid in interpretation when FNA alone is performed. Our pathologists have extensive experience in cytopathology but in this study there were no immunostains used in the cytologic evaluation; because the pathologists knew that additional tissue would be examined by core biopsy, a factor that may have decreased the sensitivity of FNA.

As demonstrated by the core negative/FNA positive cases, care must be taken to be certain that the core specimen is being taken from the node; the node may be more difficult to visualize due to its depth and is frequently very mobile, making the core biopsy procedure quite challenging. Obtaining more than one core sample should insure a greater chance of obtaining an adequate specimen, as shown by the trend (albeit statistically not significant) for increased sensitivity with a greater number of core passes. Obtaining one core with a 14 g device was the least sensitive technique in this study and would not be recommended. If it is not certain that adequate cores were obtained, the radiologist should perform FNA of the node or take additional cores. In either case, samples should be taken from the node's cortex, where the metastatic cells would lodge, avoiding the hilus where the vascular supply to the node is located.

Both FNA and core biopsy (excluding the insufficient cores of ill-defined nodes) were least sensitive when the node appearance was least abnormal. This can be due to difficulty in choosing the appropriate node for sampling or due to smaller metastatic deposits in the sampled node. In 4 of the 5 cases that were both core and FNA negative, the nodes had a normal shape, visible hilus, and cortical thickness of 2.1 to 4 mm.

In our study core biopsy had no more morbidity than FNA, even with the largest gauge device. Use of a biopsy device with a nonthrow option should diminish the chance of vascular injury. However, patients whose suspect node was immediately adjacent to a vessel or very deep and difficult to access were not asked to participate in the study and hence were not subjected to core biopsy. Despite the statistically significant difference we observed in the number of patients reporting pain being greater during core than FNA, the majority of patients tolerated the pain equally well during both procedures, and we do not believe this should be a factor in deciding which procedure to perform.

Limitations of our study included its small size, in particular, the small size of subgroups of needle types and number of samples obtained. Although there may have been some selection bias due to excluding patients with nodes not suited to core biopsy, the aim of the study was to compare the two methods when both were possible. In all cases, the core biopsy was performed after the FNA, with additional lidocaine, which may have minimized the pain associated with core biopsy. FNA was always performed first because of concern that core biopsy might cause sufficient bleeding to have to abort the second sampling procedure, but bleeding was not a significant problem. A large fraction of patients underwent neoadjuvant chemotherapy, which was not predicted at the time of initiation of the study. This could have rendered some patients node negative that were initially node positive, but there were only 7 that were node negative after chemotherapy and node negative by both core and FNA. If FNA and core were both falsely negative, there would be a similar reduction in sensitivity for each method. Unsuccessful neoadjuvant chemotherapy could result in nodes initially negative becoming positive. However, the 5 patients which were both FNA and core negative and with positive nodes at surgery did not have neoadjuvant chemotherapy. Table 2 shows the sensitivities of core biopsy and FNA in patients chosen to receive chemotherapy to be better than in those going directly to surgery, which is likely a reflection of the fact that the neoadjuvant group had more abnormal appearing nodes with thicker cortices.

Our study began before the ACOSOG Z0011 trial [8] that reported in 2010 no statistically significant differences in local or regional recurrence after median follow-up of 6.3 years between those randomized to sentinel node dissection alone versus completion axillary dissection in patients with clinically negative axillae and T1 or T2 invasive breast cancers treated with lumpectomy and radiation and 1 or 2 positive sentinel nodes. As a result of this trial, surgeons indicated that they would perform sentinel node biopsy even after a positive percutaneous node biopsy to determine if only one or two nodes were positive rather than perform axillary dissection in patients with tumors less than 5 cm. Consequently some surgeons have requested that radiologists not biopsy suspicious nodes in these patients. However, the Z0011 patient population had a low tumor burden with median tumor sizes of 1.6 and 1.8 cm, and a high percentage of micrometastases and solitary positive nodes, suggesting that their outcome may be different than those with positive percutaneous node biopsies. Although the Z0011 trial results have called into question the value of preoperative tissue sampling of axillary nodes in a selected population, the preoperative detection of metastatic adenopathy at the time of breast cancer diagnosis will continue to be helpful in management of patients with a larger tumor burden and allow many women to have axillary dissection at the time of definitive breast surgery, sparing them an unnecessary sentinel node procedure. For patients who will undergo neoadjuvant chemotherapy, the Z0011 results do not apply; percutaneous axillary node sampling will aid in proper staging prior to treatment.

The decision to perform core biopsy versus FNA should be based on the pathologist's experience in interpreting cytology and the accessibility of the lymph node. Core biopsy should be considered if the node is clearly imaged and readily accessible. Fine needle aspiration is a good alternative to core biopsy when a smaller needle is desired due to node location or other patient related factors. Care should be taken to obtain sufficient material for cytologic or histopathologic evaluation.

## Figures and Tables

**Table 1:** Features of population of 95 patients in study cohort.

	Number of patients Node positive	Number of patients Node negative	Number of patients Neoadjuvant therapy	Number of patients No chemotherapy	Other*
Number of patients	70	25	47	44	4
Tumor size by imaging					
5 mm–1 cm	4 (6)	6 (24)	3 (6)	7 (16)	0 (0)
>1 cm-2 cm	19 (27)	7 (28)	12 (26)	13 (30)	1 (25)
>2 cm–5 cm	36 (51)	11 (44)	25 (53)	20 (45)	2 (50)
>5 cm	11 (16)	1 (4)	7 (15)	4 (9)	1 (25)
Hilus present	51 (73)	22 (88)	34 (72)	36 (82)	3 (75)
Hilus absent	19 (27)	3 (12)	13 (28)	8 (18)	1 (25)
Cortex					
2 mm	1 (1)	0 (0)	1 (2)	0 (0)	0 (0)
2.1–4 mm	13 (19)	18 (72)	9 (19)	22 (50)	0 (0)
4.1–6 mm	20 (29)	3 (12)	13 (28)	9 (20)	1 (25)
>6 mm	36 (51)	4 (16)	24 (51)	13 (30)	3 (75)
Shape					
Normal	21 (30)	9 (36)	13 (28)	17 (39)	0 (0)
Focal bulge	20 (29)	12 (48)	14 (30)	10 (23)	3 (75)
Round	23 (33)	4 (16)	16 (34)	15 (34)	1 (25)
Ill-defined	6 (9)	0 (0)	4 (8)	2 (4)	0 (0)
Number of suspicious nodes					
1	31 (44)	14 (56)	18 (38)	24 (55)	3 (75)
2	16 (23)	3 (12)	11 (23)	10 (23)	0 (0)
3	10 (14)	5 (16)	7 (15)	5 (11)	1 (25)
4 or more	13 (19)	3 (12)	11 (24)	5 (11)	0 (0)

*Note.* Numbers in parentheses are percentages. Percentages are rounded.

*Other: 4 patients were both core and FNA positive but did not have axillary surgery.

**Table 2:** Sensitivity of core biopsy versus FNA for detection of 70 patients with Axillary node metastases.

Factor	*N*	Core detected *N* (%)	FNA detected *N* (%)	Difference in sensitivities (core-FNA)	Discordant	*P* value
Overall	**70**	**61 (87.1)**	**55 (78.6)**	**8.6%**	**14**	**0.18**
Number of core passes						
1 pass	14	11 (78.6)				
>1	56	50 (89.3)				
Core needle size						
12 g	9	8 (88.9)				
1 core pass	8	7 (87.5)				
>1 core pass	1	1 (100)				
14 g or 18 g	61	53 (86.9)				
1 core pass	6	4 (66.7)				
>1 core pass	55	49 (89.1)				
Number of FNA entries						
1 entry	60		46 (76.7)			
>1	10		9 (90)			
FNA needle size						
21 g	53		41 (77.4)			
1 FNA entry	46		35 (76.1)			
>1 FNA entry	7		6 (85.7)			
25 g	17		14 (82.4)			
1 FNA entry	14		11 (78.6)			
>1 FNA entry	3		3 (100)			
Hilus						
Present	51	44 (86.3)	36 (70.6)	15.7%	12	
Absent	19	17 (89.5)	19 (100)	−10.5%	2	
Cortex						
2 mm	1	1 (100)	0 (0)	100.0%	1	
2.1–4 mm	13	8 (61.5)	7 (53.8)	7.7%	3	
4.1–6 mm	20	18 (90)	15 (75)	15.0%	5	
>6 mm	36	34 (94.4)	33 (91.7)	2.8%	5	
Shape						
Normal	21	16 (76.2)	11 (52.4)	23.8%	5	
Focal bulge	20	19 (95)	16 (80)	15.0%	5	
Round	23	22 (95.7)	22 (95.7)	0.0%	2	
Ill-defined	6	4 (66.7)	6 (100)	−33.3%	2	
Number of suspicious nodes						
1	31	26 (83.9)	23 (74.2)	9.7%	7	
2	16	13 (81.3)	11 (68.8)	12.5%	4	
3	10	10 (100)	9 (90)	10.0%	1	
4 or more	13	12 (92.3)	12 (92.3)	0.0%	2	
Tumor size by imaging						
5 mm–1 cm	4	4 (100)	2 (50)	50.0%	2	
>1 cm-2 cm	19	16 (84.2)	14 (73.7)	10.5%	4	
>2 cm–5 cm	36	31 (86.1)	31 (86.1)	2.8%	5	
>5 cm	11	9 (81.8)	8 (72.7)	9.1%	3	
Chemotherapy status						
No chemotherapy	26	20 (76.9)	17 (65.4)	11.5%	5	
Neoadjuvant	40	37 (92.5)	34 (85.0)	7.5%	9	
Other*	4	4 (100)	4 (100)	0.0%	0	

*Other: 4 patients were both core and FNA positive but did not have axillary surgery.
